# Evaluating Attitudes of First-Year Residents to Shared Decision Making

**DOI:** 10.3885/meo.2008.Res00276

**Published:** 2008-07-10

**Authors:** Jon G. Caldwell

**Affiliations:** Department of Psychiatry, University of California at Davis, Davis, California USA

**Keywords:** Decision Making, Interviews/methods, Patient Participation/methods, Physician-Patient Relations, Patient Participation/psychology, Physician's Role/psychology, Communication, Physicians, Education, Training, Internship and Residency, Internship, Medical Residency

## Abstract

**Objective:**

Shared decision making (SDM) is recognized as an ideal model of patient-physician interaction, yet clinical application occurs infrequently. The current study evaluated attitudes of first-year residents to identify potential barriers and opportunities regarding SDM.

**Methods:**

A total of 70 residents attending orientation at the University of Utah completed a questionnaire that elicited their understanding of SDM, perceptions about the importance of SDM, confidence in utilizing SDM, and reasons for lacking confidence.

**Results:**

Most residents reported no prior SDM education (N = 42, 60%) or training (N = 46, 66%), yet 67 (96%) of them could recognize it in a clinical vignette. Using a Likert scale, the majority of residents (91% to 99%) attributed importance to SDM principles, and most (79% to 90%) indicated confidence in applying them. Lack of training was reported as a barrier by 40 (57%) residents.

**Conclusions:**

A minority of residents reported formal education or training in SDM, yet the vast majority recognized and valued the model. A large percentage of residents expressed confidence in their abilities to incorporate SDM into patient care, but many also identified a need for more education and training.

Despite extraordinary advances in scientific knowledge and technology, the patient-physician relationship remains a vital component of the medical encounter. Social, ethical, and medicolegal trends have led to a growing acceptance of “patient-centered” approaches to health care delivery.^1^ The advancement of guidelines regarding “informed consent” represents an important legal aspect of medical decision-making.^2^ However, it is increasingly recognized that in addition to sharing information necessary for consent, physicians have an ethical obligation to actively involve patients in a collaborative, two-way process regarding their medical treatment.^3^ Traditional “paternalistic” models of medical decision-making, in which the physician makes unilateral decisions on behalf of the patient, are now seen as suboptimal in most treatment settings.^4^ Shared decision making (SDM) has emerged as an important model of interactive communication that works to simultaneously engage patient and physician in all stages of the medical decision-making process.^5^

While a number of models have been proposed to enhance patient-physician communication and medical decision-making, the present study focuses on an influential model put forth by Charles et al.^5^ This model identifies four key aspects of SDM: (1) both the patient and the doctor are involved in the decision-making process, (2) both parties share information, (3) both parties take steps to build a consensus about the preferred treatment and (4) a mutual agreement is reached on the treatment to implement. Subsequent research has identified other important elements of SDM, including fostering a therapeutic relationship, understanding the patient's preferences for role in decision making, discussion of the problem and nature of the decision, identification of evidence-based choices and their bearing on the patient's values and lifestyle, negotiating a decision in partnership with the patient, agreeing on an action plan, and checking for mutual understanding.^6^,^7^

While there is evidence that employing SDM is ethically sound, can enhance patient-physician interactions, and may improve healthcare outcomes,^8^–^10^ the integration of these principles into clinical practice occurs infrequently.^11^,^12^ Several challenges to the successful adoption of SDM have been identified, including system-related issues (i.e., time constraints), patient-related issues (i.e., variability in desired level of participation), and practitioner-related issues (i.e., lack of training).^13^ There is evidence that practicing physicians are receptive to SDM and willing to acquire the relevant skills,^14^ yet little is known about the attitudes of physicians in training. The purpose of this study was to evaluate the attitudes of first-year residents in order to identify limitations and opportunities regarding the education and practice of SDM in patient care.

## Methods

**Study Sample**-Data collection procedures were reviewed by the University of Utah Institutional Review Board and the office of Graduate Medical Education. A questionnaire was administered to first-year residents during their orientation session for residency training at the University of Utah Health Sciences Center in July of 2005. The sample included M.D. and D.O. physicians from 43 medical schools entering residency training programs in 16 different medical specialties.

**Measures**-The questionnaire used in this study was developed by the principal investigator with input from other researchers attending the Third International Shared Decision Making Conference held at the University of Ottawa, Canada in June, 2005. The questionnaire was pilot-tested among a group of residents with demographic variables similar to study participants. It contained 41 items and was divided into 5 main sections: 1) Resident characteristics (sex, age, race, degree type, medical specialty, and medical school) 2) Knowledge of SDM (2 questions asked whether residents had received education or training in SDM, 1 open-ended question asked residents to describe what they thought SDM meant, and 1 multiple choice question asked residents to choose one of four short vignettes that most closely reflected an SDM approach). Vignettes were adopted with permission from Charles et al.^15^ 3) Importance of SDM (15 items dealing with medical decision making were tied to a five-point Likert scale anchored at both ends by “Not Important” and “Very Important”. Items 1-5 were considered general aspects of patient care, whereas items 6-15 were considered specific elements of SDM – adopted and altered with permission from Elwyn et al.^16^) 4) Confidence in Implementing SDM; the same 15 items were used again with a different five-point Likert scale anchored at both ends by “Not Confident” and “Very Confident”. 5) Potential Barriers; 1 multiple-choice question asked residents to identify a reason that best explained their lower levels of confidence in being able to implement SDM.

**Design and Data Collection**-In this cross-sectional study, a questionnaire was distributed to all first-year residents who attended the orientation seminar. After a thorough description of the study, written informed consent was obtained. Adequate time was provided, and most residents completed the questionnaire in less than 15 minutes. Data were analyzed descriptively by individual item using percentage distributions.

## Results

**Resident Characteristics**-A total of 86 first-year residents attended at the orientation, and 70 (81%) returned questionnaires suitable for data analysis. Demographic data were not available for those residents who chose not to submit a questionnaire, so statistical comparison of questionnaire responders and non-responders was not possible. Of the respondents, 41 (59%) were male and 29 (41%) were female; 57 (81%) were Caucasian, 9 (13%) were Asian, 2 (3%) were Hispanic/Latino, 1 (1%) was African American, and 1 (1%) self-identified as Other. In terms of age, 63 (90%) residents were between 26-35, 5 (7%) were between 18-25, 1 (1%) was between 36-45, and 1 (1%) was over 45. Regarding training, 67 (96%) residents had M.D. degrees and 3 (4%) had D.O. degrees. Sixteen different medical specialties were listed, with Internal Medicine (N = 25, 36%) being most common; 43 different medical schools were represented, with the largest cohort (N = 7, 10%) coming from the University of Utah.

**Knowledge of SDM**-Among the first-year residents evaluated in this study, 42 (60%) had never received education about SDM and 46 (66%) had never received training on implementing SDM in clinical practice. Nevertheless, when asked the meaning of SDM, 44 (61%) residents gave an answer that was considered congruent with the definition used in this study. Furthermore, a majority of residents (N = 67, 96%) were able to correctly identify a vignette illustrating SDM principles when it was presented in multiple choice fashion with three other vignettes reflecting different approaches (i.e., paternalism, informed, and some sharing-information only).

**Importance of SDM**-As expected, nearly all residents (69 – 70 or 99 – 100%) found items 1-5, considered general aspects of patient care, to be important concepts in clinical practice. While some of the items numbered 6-15 were identified as relatively less important than items 1-5, the vast majority of residents (64 – 69 or 91 – 99%) identified items specific to SDM as important elements in patient care. Of interest, the fewest number of residents (N = 64, 91%) attributed high levels of importance to identifying the patient's desired level of involvement in decision making process. Results are summarized in Table 1.

**Table 1:** Percentage of first-year residents who reported high levels of importance and confidence (N = 70)

	High levels of importance^b^^c^		High levels of confidence^d^^e^	
Questionnaire Items^a^	N	%	N	%
1. Develop a strong therapeutic relationship with the patient	69	99	62	89
2. Gather necessary information from the patient about the problem	70	100	54	77
3. Consolidate the gathered information into ideas about diagnosis	70	100	40	57
4. Determine an appropriate treatment plan for the identified problem	70	100	32	46
5. Obtain informed consent prior to treatment or procedures	69	99	58	83
6. Actively encourage patient participation throughout the encounter	66	94	63	90
7. Clearly identify problems that require a decision making process	68	97	47	67
8. Identify desired level of involvement in decision making process	64	91	46	66
9. Portray more than one treatment option, including “no action”	69	99	55	79
10. Discuss evidence-based treatment options and pros/cons of each	66	94	31	44
11. Explore patient's ideas, fears, and expectations of various options	68	97	56	80
12. Assess patient's understanding and comfort with decision process	67	96	57	81
13. Explore patient beliefs and values associated with their preference	66	94	55	79
14. Negotiate a treatment decision in partnership with the patient	69	99	56	80
15. Agree on an action plan and make arrangements for follow-up	67	96	57	81

a. Items numbered 1-5 were considered general aspects of patient care, whereas items numbered 6-15 were considered specific elements of SDM.

b. The actual instructions were: “Please rate how important you think it is for a physician to incorporate each of the following concepts into patient care.”

c. Level of importance was determined by numbering and collapsing the five-point Likert scale categories (1 = “Not Important”; 5 = “Very Important”) such that responses 4 and 5 were added together and operationalized as the percentage of residents that ascribed high levels of importance to incorporating each of the items into patient care.

d. The actual instructions were: “Please rate how confident you feel in your current abilities to incorporate each of the following concepts into patient care.”

e. Level of confidence was determined by numbering and collapsing the five-point Likert scale categories (1 = “Not Confident”; = “Very Confident”) such that responses 4 and 5 were added together and operationalized as the percentage of residents who reported high levels of confidence in their abilities to incorporate each item into patient care.

**Confidence in Implementing SDM**-Regarding general aspects of patient care (items 1-5), a relatively large percentage of residents felt confident in their abilities to develop a therapeutic relationship (N = 62, 89%) and gather necessary information about the problem (N = 54, 77%). However, residents were less confident in their abilities to consolidate information into ideas about diagnosis (N = 40, 57%) and to determine an appropriate treatment plan (N = 32, 46%). In contrast, on 7 of the 10 items numbered 6-15, greater than three quarters of residents expressed confidence in their abilities to incorporate SDM principles into patient care (55 – 63 or 79 – 90%). Of note, the fewest number of residents (N = 31, 44%) expressed high levels of confidence in discussing the pros and cons of evidence-based treatment options. Results are summarized in Table 1.

**Potential Barriers**-When first-year residents were asked to explain why they expressed lower levels of confidence in being able to implement SDM into patient care, 40 (57%) identified a need for more education and training, 4 (6%) considered system issues a barrier, and 3 (4%) felt as though patients were unwilling or unable to engage in SDM. The remainder of residents (N = 23, 33%) did not identify any particular barriers and felt confident in their abilities to practice SDM.

## Discussion

While less than half of first-year residents in this sample had received formal education or training in SDM, the majority of residents were able to recognize and define the model in basic terms. These results may reflect the fact that this relatively young group of physicians has been exposed to contemporary trends towards increased patient involvement in the medical encounter. Similar to published findings among samples of first-year residents^17^ and practicing physicians,^18^ residents in this study indicated strong support for patient involvement in medical decision making. These findings suggest that physicians today are increasingly unencumbered by notions of “paternalism” and seem to have an awareness of and solid appreciation for SDM principles.

Consistent with recent data from a large nationally representative sample of physicians,^19^ the majority of first-year residents in this study expressed high levels of confidence with an SDM approach. While these findings are encouraging, researchers have cautioned that physicians may overestimate their abilities to practice certain principles of SDM.^20^ Perhaps illustrating this point, 80% of first-year residents in the current study expressed confidence with the SDM item “negotiate a treatment decision in partnership with the patient”, yet only 46% indicated confidence on a similar general patient-care item, “determine an appropriate treatment plan for the identified problem”. Additionally, 85% of respondents felt confident in their ability to obtain informed consent, yet only 44% felt confident in discussing evidence-based treatment options, an element considered by most researchers as critical to the informed consent process.^2^ These data may corroborate previous findings regarding physicians’ propensity to overestimate their abilities in practicing SDM.

Regarding possible barriers, of those residents who reported less confidence in their abilities to implement SDM, 85% identified a need for more education and training, a view which has been expressed by physicians in other studies.^21^ First-year residents in this study attributed considerable value to SDM principles and expressed a willingness to receive more education and training on the subject. Moreover, compared to a sample of practicing physicians,^13^ residents were less concerned about systemic issues and patient variables being barriers to the practice of SDM. There is evidence to suggest that utilization of SDM in clinical practice may be enhanced by intervening before patient-physician interaction patterns have become well-established.^20^ Therefore, physicians in training may play a vital role in understanding the limitations and opportunities for SDM in the health care delivery system.

One important contribution of this study is the unique sample. Residents’ attitudes have rarely been studied in the literature on SDM but may be valuable markers in determining current trends and future directions. One limitation is that the questionnaire used in this study has not been formally validated, although it was heavily influenced by measures proven reliable and valid in other studies.^15^,^16^ Further research might evaluate how SDM is currently taught and practiced in medical schools and residency training programs. Also, the crucial questions regarding the discrepancy between acknowledgment of SDM as an important model and the general lack of its incorporation into patient care might be addressed further by evaluating the attitudes, education, and training of medical residents.
